# Dietary Sodium Lactate Alleviates Ammonia Stress‐Induced Growth Impairment, Oxidative Damage and Metabolic Disorder in Juvenile Yellow Catfish

**DOI:** 10.1155/anu/7834375

**Published:** 2026-07-08

**Authors:** Ruiqiong Zhang, Ming Li, Yanjie Tang, Ying Yan, Xuan Chen, Haibo Jiang, Muzi Zhang

**Affiliations:** ^1^ School of Marine Sciences, Ningbo University, Ningbo, 315211, China, nbu.edu.cn; ^2^ College of Animal Science, Guizhou University, Guiyang, 550025, China, gzu.edu.cn

**Keywords:** chronic ammonia stress, growth, sodium lactate, yellow catfish

## Abstract

This study aimed to analyze the beneficial impacts of dietary sodium lactate (SLA) on growth, metabolism, and ammonia resistance in juvenile yellow catfish (*Pelteobagrus fulvidraco*) under chronic ammonia stress. In an 8‐week experiment, 360 healthy juveniles (1.64 ± 0.03 g) were assigned to 4 groups (3 replicates, 30 fish/replicate), fed either a basal diet or a 1.00% SLA‐supplemented diet with or without 2.5 mg/L total ammonia nitrogen (T‐AN) exposure. The results showed that dietary SLA supplementation effectively ameliorated growth inhibition and impaired feed efficiency caused by chronic ammonia stress. At the digestive level, SLA supplementation significantly enhanced the activities of intestinal lipase and pepsin, with no significant change in amylase activity. In addition, dietary SLA positively regulated serum biochemical profiles, improved hepatic antioxidant capacity, and alleviated oxidative damage induced by long‐term ammonia exposure. At the molecular level, SLA modulated the expression of hepatic glucose metabolism‐related genes, reversed the inhibitory effect of chronic ammonia stress on muscle growth‐related gene expression, and downregulated the growth‐suppressive gene *mstn*. Collectively, dietary SLA can effectively mitigate the adverse effects of chronic ammonia stress on juvenile yellow catfish by improving digestive function, regulating metabolic homeostasis, and alleviating hepatic oxidative damage and ammonia toxicity. This study supports the application of SLA as a functional feed additive in aquaculture.

**Summary**

• First confirmation that sodium lactate (SLA) mitigates chronic ammonia stress injury in yellow catfish.
• Clarified that SLA enhances the digestive function and ammonia detoxification capacity of yellow catfish.
• Revealed that SLA activates the antioxidant system to alleviate hepatic oxidative damage.
• Elucidated candidate molecular pathways underlying SLA‐regulated energy metabolism and muscle growth.
• Provided a novel nutritional regulation strategy for ammonia stress resistance in aquaculture.

## 1. Introduction

Yellow catfish (*Pelteobagrus fulvidraco*) is an economically important freshwater aquaculture species in China and other Asian regions. Owing to its tender meat, high nutritional value, and few intermuscular bones, it holds a crucial position in aquaculture [1]. However, intensive and high‐density farming models lead to excessive accumulation of metabolic wastes in the culture water, resulting in ammonia stress [2]. Chronic ammonia exposure can cause nitrogen metabolism disorders, insufficient energy supply, impaired intestinal barrier function, and oxidative damage to key tissues (e.g., the liver and intestine) in yellow catfish, ultimately leading to growth retardation and reduced survival rates, which inflict significant economic losses on the aquaculture industry [3]. Therefore, the development of safe and effective regulatory strategies to mitigate the detrimental impacts of prolonged ammonia stress on juvenile yellow catfish has emerged as an urgent priority to be addressed in contemporary aquaculture research [4, 5].

In the past few years, studies on the microbiota–host interaction mechanism have provided new insights into improving the stress resistance of fish [6]. Intestinal probiotics enhance fish ammonia tolerance through multiple mechanisms, including synthesizing key metabolic intermediates to promote host ammonia detoxification, degrading waterborne ammonia, and activating host antioxidant and immune pathways [1]. For example, *Cetobacterium somerae* promotes urea production in yellow catfish, while *Bacillus subtilis* DM115 improves koi survival under ammonia stress [7, 8]. Although the above studies have demonstrated that intestinal probiotics can enhance the stress resistance of aquatic animals through multiple metabolic regulation pathways, the practical application of such viable bacteria‐dependent probiotics remains constrained by factors such as feed processing conditions and intestinal colonization efficiency, making it difficult to ensure the consistency of their effects [9, 10].

To overcome these limitations, microbial metabolite substitutes (also termed postbiotics) have emerged as a promising alternative to live probiotics in aquaculture. Unlike live microorganisms, these well‐defined compounds are thermostable during feed extrusion processing, do not require intestinal colonization to exert biological effects, and deliver consistent and reproducible results [11]. Short‐chain fatty acid derivatives, a major class of postbiotics, have been widely studied for their beneficial effects on fish growth and stress resistance. Sodium butyrate improves growth and ammonia tolerance in yellow catfish by modulating the intestinal microbiota and digestive function, while sodium propionate enhances antioxidant capacity and reduces inflammation in rainbow trout [3, 12]. However, existing research has mainly focused on acetate, propionate, and butyrate salts, while the role of lactate‐derived microbial metabolites in alleviating ammonia toxicity in fish remains largely unexplored. As a representative lactic acid metabolite, sodium lactate (SLA) not only shares the common advantages of postbiotics (thermal stability, no need for intestinal colonization, and consistent efficacy) but also has a unique advantage: it may serve as an energy substrate to participate in glycolysis and the tricarboxylic acid (TCA) cycle, rapidly meeting the elevated energy demands under stress conditions [13].

In our prior research, we found that the abundance of *Lactobacillus amylolyticus*, a lactic acid‐producing bacterium, was significantly increased in the intestine of yellow catfish under chronic ammonia stress, and its primary metabolite, lactic acid, promoted muscle cell proliferation and improved ammonia tolerance in vitro. As a stable and bioavailable sodium salt form of lactic acid, SLA is more suitable for aquafeed supplementation. Nevertheless, the protective function and molecular mechanism of SLA against ammonia toxicity in fish remain unclear. Therefore, the objective of this study was to investigate the protective effects of dietary SLA on juvenile yellow catfish under chronic ammonia stress and its underlying molecular mechanisms.

## 2. Materials and Methods

### 2.1. Ethical Statement

The Animal Care Advisory Committee of Ningbo University granted approval for all management conditions and experimental protocols. All experimental procedures covered by these protocols were performed in strict accordance with Ningbo University’s Guidelines for the Care and Use of Laboratory Animals and under the formal authorization of the official animal use license Number SYXK (ZHE 2012‐011012).

### 2.2. Experimental Diets

In accordance with the nutritional requirements of the experimental fish, two isonitrogenous and isolipidic test diets were formulated with the addition of 0.00% and 1.00% SLA (purity ≥ 99%, Zhenhai Baichuan, Ningbo) to the basal diet for yellow catfish [13]. All raw materials were first ground into a fine powder and passed through an 80‐mesh screen, after which they were mixed sequentially with fish oil, soybean oil, and purified water until a uniform homogeneous mixture was formed. The resulting mixture was processed into a 3 mm diameter pellet feed using an F‐26II twin‐screw extrusion and pelleting machine (South China University of Technology, Guangzhou). The prepared feed pellets were air‐dried naturally, then sealed, and kept at a constant temperature of −20°C until subsequent experiments. The detailed feed formula and the proximate nutrient composition of the final diets are presented in Table 1.

**Table 1:** The experimental diets composition and proximate composition (%, dry matter).

Ingredients	Dietary supplementation levels of sodium lactate	
	CON	SLA
Fish meal	20.00	20.00
Soy protein	16.00	16.00
Corn gluten meal	8.00	8.00
Soybean meal	22.00	22.00
Rapeseed meal	1.30	1.30
Cottonseed meal	1.30	1.30
Fish oil	3.20	3.20
Soybean oil	3.20	3.20
Starch	20.50	20.50
Zeolite powder	1.50	0.50
Vitamin premix^a^	0.50	0.50
Mineral premix^b^	0.50	0.50
Monocalcium phosphate	1.00	1.00
Sodium carboxymethyl cellulose	1.00	1.00
Sodium lactate^c^	0.00	1.00
Proximate composition		
Crude protein	39.75	39.78
Crude lipid	8.86	8.89

^a^Vitamin premix (per kg feed): VA 8 000 000 IU; VD 2 000 000 IU; VE 5 000 UI; VK 1000 mg; VB1 1500 mg; VB2 1500 mg; VB6 800 mg; VB12 20 mg; Niacinamide 400 mg; Calcium pantothenate 25 mg; Folic acid 25 mg; Biotin 8 mg; Inositol 100 mg.

^b^Mineral premix (per kilogram of feed): MnSO_4_ · H_2_O 50 mg; KI 100 mg; CoCl_2_ (1%) 100 mg; CuSO_4_ · 5H_2_O 20 mg; FeSO_4_ · ⋅H_2_O 260 mg; ZnSO_4_⋅H_2_O 150 mg; Na_2_SeO_3_ (1%) 50 mg.

^c^Sodium lactate was purchased from Ningbo Zhenhai Baichuan Biotechnology Co., Ltd., Ningbo.

### 2.3. Fish and Feeding Trial

Healthy yellow catfish individuals were obtained from Jiaxing, China, and subjected to a 14‐day laboratory acclimation period prior to the formal trial. In total, 360 robust fish with a consistent initial body mass (1.64 ± 0.03 g) were chosen and randomly allocated into 12 aquaria. These fish were then assigned to four treatment groups, with each aquarium defined as one experimental unit. Each group contained three replicate aquaria, and each aquarium held 30 fish. Two total ammonia nitrogen (T‐AN) levels were established: 0 mg/L T‐AN (control) and 2.5 mg/L T‐AN (chronic stress group). This concentration was selected based on our systematic 56‐day chronic toxicity study in yellow catfish [14], which showed that 1.25 mg/L T‐AN was the upper limit for normal growth, while 2.5 mg/L T‐AN induced consistent sublethal effects with <10% mortality. This corresponds to ~0.03 mg/L unionized ammonia, 22.7‐fold lower than the 96‐h LC_50_ under our experimental conditions. At each ammonia level, fish were fed twice daily with one of the two experimental diets at 05:00 and 17:00, respectively. The four experimental groups were designated as follows: CON group (0 mg/L T‐AN, basal diet), AM group (2.5 mg/L T‐AN, basal diet), SLA group (0 mg/L T‐AN, basal diet + 1.00% SLA), and SLA + AM group (2.5 mg/L T‐AN, basal diet + 1.00% SLA). The total experimental period was 56 days. Aeration was provided via air stones, and one‐third of the water volume in each glass aquarium was replaced daily. Ammonia nitrogen levels were measured using a YSI ProPlus water quality multiprobe instrument (YSI Inc., USA). Water temperature was maintained at 29–30°C, dissolved oxygen was kept above 6.80 mg/L, and nitrate levels remained below 0.1 mg/L. A natural photoperiod was applied throughout the experimental period.

### 2.4. Sample Collection

At the conclusion of the experiment, all fish were fasted for 24 h and anesthetized with MS‐222 (120 mg/L) before counting and weighing. For growth performance and body composition analysis, each aquarium was used as one biological replicate. Three fish were randomly chosen from each aquarium and kept at −20°C for the determination of the whole‐body proximate composition. Meanwhile, three additional individuals from each tank were subjected to blood extraction through the caudal vein. Blood samples were centrifuged at 836 ×*g* for 10 min at 4°C to acquire the serum, which was preserved at −20°C until subsequent analysis. Following blood sampling, the liver, intestine, and muscle tissues were dissected from these fish and frozen at −80°C for later biochemical and molecular analyses. All serum, tissue biochemical indicators, and gene expression levels were measured using individual fish samples. Furthermore, an additional three fish from each tank were dissected for the collection of hepatic samples. The liver weight was recorded to calculate the hepatosomatic index.

### 2.5. Body Composition Analysis

Proximate compositions including crude protein, crude lipid, moisture, and ash in both experimental diets and whole fish samples were analyzed in accordance with the standard protocols recommended by the AOAC [15]. The moisture level was determined by drying the samples in a drying oven at 105°C until constant weight was achieved. An FP‐528 automatic protein analyzer (Leco Corporation, USA) was used to determine the crude protein content. The crude lipid content was measured using a 2055 automatic Soxhlet extraction system (Foss, Sweden). For ash determination, precarbonated samples were placed in a muffle furnace at 550°C and continuously calcined until reaching a constant weight.

### 2.6. Biochemical Analysis

After collection from the caudal vein, the blood samples were centrifuged at 836 *g* for 10 min at 4°C to isolate the serum. The resulting serum was used to determine the levels of total protein (TP), triglyceride (TG), total cholesterol (TC), alanine aminotransferase (ALT), aspartate aminotransferase (AST), albumin (ALB), blood urea nitrogen (BUN), lactate, pyruvate, serum ammonia (SA), and the activity of lactate dehydrogenase (LDH).

Liver samples were mixed with nine volumes (w/v) of ice‐cold phosphate‐buffered saline (PBS), then homogenized completely using a homogenizer (IKA, Germany). After centrifugation at 8000 ×*g* for 10 min at 4°C, the collected supernatant was used for the measurement of total antioxidant capacity (T‐AOC), superoxide dismutase (SOD), and catalase (CAT) activities, as well as glutathione (GSH) and malondialdehyde (MDA) contents.

After homogenization on ice in nine volumes (w/v) of prechilled PBS, the intestinal tissue homogenate was centrifuged at 8000 ×*g* for 10 min at 4°C, with the supernatant collected for the determination of lipase, amylase, and pepsin activities.

Biochemical analyses in this research were conducted using commercial assay kits supplied by Nanjing Jiancheng Bioengineering Institute (China), with all procedures strictly performed following the manufacturer’s protocols.

### 2.7. Total RNA Isolation, cDNA Preparation and Quantification of Relative mRNA Expression

Total tissue RNA was extracted using the RNAiso kit (Takara, China), and first‐strand cDNA was prepared with the PrimeScript RT reagent kit (Takara, China). Quantitative real‐time PCR was conducted on a LightCycler 480 II real‐time PCR system (Roche, Switzerland) using SYBR Premix Ex Taq (Takara, China). The total reaction volume of 20 μL consisted of 8.8 μL ddH_2_O, 10 μL 2× SYBR Premix Ex Taq, 0.4 μL reverse primer, 0.4 μL forward primer, and 0.4 μL cDNA. The amplification protocol comprised an initial denaturation at 95°C for 30 s, followed by 40 cycles of denaturation at 94°C for 5 s, annealing at 55°C for 15 s, and extension at 72°C for 10 s. Table 2 lists all the gene‐specific primers used in this study. Each reaction was conducted in triplicate. The 2^(‐ΔΔCT) method was employed to quantify gene expression levels using β‐actin and GAPDH as the reference genes.

**Table 2:** All the primer sequences used in this experiment.

Gene	Primer sequence (5′‐3′)	Size (bp)	Accession number
*igf-1*	F: GCACAACCGTGGCATTGTAG	135	XM_027160377.2
	R: GACGTGTCTGTGTGCCGTT		
*mek*	F: CAGCAGCAGCAAGCGTTACAG	224	XM_027144355.2
	R: CGGAAGTGCAACTCCACCTCA		
*erk*	F: ACCGTGCTCCAGAGATCATGCT	237	XM_027161071.2
	R: TGGGCTTTGGTGGGAGAGACTG		
*mstn*	F: GCGCACCAAGAGAGAATCAG	125	XM_027171347.2
	R: AGCGTTTCGGGGCAATAATC		
*myod*	F: TATTCCGTTCCCCATCCCCT	208	XM_027173047.2
	R: TTTACACGCCCACAGGAGAC		
*myog*	F: ACCCGTACTTTTTCCCCGAA	129	XM_027144245.2
	R: CATCCCCACATAGCCCTACC		
*myf5*	F: GGCTAGAGAAGGTGAACCAC	290	XM_027155249.2
	R: CGCACTCTGACCTTCGTAAC		
*cs*	F: AACACCTTCCCAATGACCCC	280	XM_027144899.2
	R: GCCATAAGACCATCCGTGCT		
*idh1*	F: CATTACTCCTCGAACTGCCCC	123	XM_027143610.2
	R: TCCACAACAGATCCAGCCTTG		
*sdha*	F: CAGCGAAGGCGAGAGGTTTA	249	XM_027163703.2
	R: AAGACTGGGATGGGGTCCTT		
*pepck*	F: AGACGGCGGACATTTTGTCT	205	XM_027155792.2
	R: AGCACCTTGTCTGGATTGCA		
*pcxa*	F: AGGTCTGCTGTTGATGGACA	194	XM_047805687.1
	R: TCTTCCAAGGACACTCGCAT		
*pcxb*	F: TCAGGAGTTTGCGAAGACCT	194	XM_047810476.1
	R: TGTGGCGTGGTTTCTCAATG		
*hk1*	F: GAGACATCATGGCCCGTTTC	180	XM_027174934.2
	R: CTTTCACTCGCAGGATTCGG		
*gk*	F: TAAAGAAGGCTGGGTGGAGG	194	XM_027156114.2
	R: CGACTGCATTGTAGAGTGGC		
*pkm*	F: CATCATGGTTGCTCGTGGAG	232	XM_027159273.2
	R: TGCAGTCTCTCCGCTTAACA		
*pfkp*	F: GCCACCAGAGGACTTTTGTG	217	XM_027149179.2
	R: CTACAATCAATGGCGCCCTC		
Internal reference gene			
*GAPDH*	F: TCTGGGGTACACAGAACACC	165	XM_027149217.1
	R: ACTAGGTCACAGACACGGTT		
*β-actin*	F: TTCGCTGGAGATGATGCT	160	XM_027148463.2
	R: CGTGCTCAATGGGGTACT		

Abbreviations: *β-actin*, actin beta*; cs*, citrate synthase; *erk*, extracellular signal‐regulated kinase; *GAPDH*, glyceraldehyde‐3‐phosphate dehydrogenase; *gk*, glucokinase; *hk1*, hexokinase 1; *idh1*, isocitrate dehydrogenase 1; *igf*‐1, insulin‐like growth factor 1; *mek*, mitogen‐activated protein kinase kinase; *mstn*, myostatin; *myf5*, myogenic factor 5; *myod*, myoblast determination protein 1; *myog*, myogenin; *pcxa*, pyruvate carboxylase a; *pcxb*, pyruvate carboxylase b; *pepck*, phosphoenolpyruvate carboxykinase; *pkm*, pyruvate kinase M; *pfkp*, phosphofructokinase platelet; *sdha*, succinate dehydrogenase complex flavoprotein subunit A.

### 2.8. Statistical Procedures

All data were first tested for normality and homogeneity of variance prior to the statistical analysis. Data in tables are expressed as the mean ± standard error of the mean (SEM). A two‐way analysis of variance (two‐way ANOVA) was used to evaluate the main effects of ammonia stress (absence vs. presence) and dietary SLA supplementation (absence vs. presence), as well as their interaction effect. The statistical model was defined as follows:

Yijk=µ+Ai+Bj+ A×Bij+eijk,

where *Y*_ijk_ represents the dependent variables (e.g., growth performance, serum biochemical indices, antioxidant enzyme activities, or gene expression levels); μ is the overall population mean; *A*_i_ is the fixed effect of ammonia stress (*i* = no ammonia stress or ammonia stress); *B*_*j*_ is the fixed effect of dietary SLA supplementation (*j* = 0 or 1.00%); (*A* × *B*)_ij_ indicates the interaction effect between ammonia stress and SLA supplementation; and *e*_ijk_ is the random error term, which follows a normal distribution with a mean of 0 and variance *σ* ^2^.

When the interaction term was significant (*p* < 0.05), simple effect analysis combined with Duncan’s multiple range test was conducted for pairwise comparisons among the four groups (CON, AM, SLA, and SLA + AM). For variables with a nonsignificant interaction (*p* ≥ 0.05), only the main effects of the two factors were interpreted. All statistical analyses were performed using SPSS 26.0 (SPSS Inc., USA), and differences were considered statistically significant at *p*  < 0.05.

The relevant formulas are as follows:

SR=FN/IN×100;

FR=100×FI/Wf+Wd+Wi/2/days;

WGR=Wf−Wi/Wi×100;

SGR=ln Wf−ln Wi/T×100;

FCR=FI/Wf−Wi;

PER=Wf−Wi/FI×CPfeed;

HSI=HW/BW×100;

VSI=VW/BW×100;

IPF=IPFW/BW×100;

CF=BW/BL3×100.

*Note*: SR: survival rate (%); FN: final number of fish; IN: initial count of fish; FR: feed rate; FI: feed ingestion; Wf: final weight; Wd: weight of mortalities; Wi: initial weight; d: day; WGR: weight gain rate (%); SGR: specific growth rate (%/d); *T*: duration of the feeding experiment (d); FCR: feed conversion ratio; PER: protein efficiency ratio; CPfeed: crude protein level in feed; HSI: hepatosomatic index (%); HW: hepatopancreas mass; BW: body mass (g); VSI: viscerosomatic index (%); VW: viscera mass; IPF: intraperitoneal fat percentage (%); IPFW: intraperitoneal fat mass; CF: condition factor (g/cm^3^); BL: body length (cm).

## 3. Results

### 3.1. Growth Performance and Body Composition

The effects of dietary SLA on the growth performance and whole‐body proximate composition of juvenile yellow catfish are summarized in Tables 3 and 4. No significant difference in IBW was observed across all treatments (*p* > 0.05), confirming uniform size distribution at the onset of the experiment. For the main effects, ammonia stress significantly decreased FBW, WGR, SGR, CF, and HSI (*p* < 0.05), and significantly increased FCR (*p* = 0.004). SR showed a decreasing trend under ammonia stress but did not reach statistical significance (*p* = 0.068). Dietary SLA supplementation significantly improved FBW, WGR, SGR, CF, and HSI (*p* < 0.05), and reduced FCR (*p* < 0.05). No significant effect of SLA on SR was detected (*p* > 0.05). FI was not significantly affected by either ammonia stress or SLA supplementation (*p* > 0.05).

**Table 3:** Effects of sodium lactate on growth performance of yellow catfish under ammonia stress.

Items	IBW (g)	FBW (g)	WGR (%)	SGR (%/day)	FCR	FI (g)	CF (g/cm^3^)	HSI (%)	SR (%)
Individual treatments									
CON	1.63	7.94	386.16	2.82	1.44	9.08	1.85	2.28	100.00
AM	1.64	7.13	335.85	2.63	1.59	8.75	1.82	2.19	94.44
SLA	1.64	8.98	447.89	3.04	1.31	9.63	1.86	2.29	98.89
SLA + AM	1.63	8.37	413.55	2.92	1.44	9.70	1.84	2.28	96.67
SEM	0.005	0.165	9.890	0.035	0.035	0.340	0.006	0.030	1.843
Main effect treatments									
No AM	1.64	8.46	417.03	2.93	1.38	9.36	1.86	2.29	99.45
AM	1.64	7.75	374.70	2.78	1.52	9.23	1.83	2.24	95.56
No SLA	1.64	7.54	361.01	2.73	1.52	8.92	1.84	2.24	97.22
SLA	1.64	8.68	430.72	2.98	1.38	9.67	1.85	2.29	97.78
*p*‐Value (two‐way ANOVA)									
AM	0.596	0.003	0.003	0.002	0.004	0.708	0.002	0.018	0.068
SLA	0.915	<0.001	<0.001	<0.001	0.004	0.060	0.046	0.018	0.771
Interaction	0.259	0.563	0.443	0.290	0.696	0.570	0.330	0.030	0.392

*Note:* Values are presented as mean ± SEM, with 3 replicate tanks per treatment. Two‐way ANOVA was applied to test the main effects of AM, SLA, and their interaction. When the interaction effect was significant (*p* < 0.05), simple effect analysis followed by Duncan’s multiple range test was performed for pairwise comparisons. Different lowercase superscript letters in the same row indicate significant differences among the four treatment groups (*p* < 0.05).

Abbreviations: AM, basal diet with 2.5 mg/L total ammonia nitrogen; CF, condition factor; CON, basal diet; FBW, final body weight; FCR, feed conversion ratio; FI, feed intake; HSI, hepatosomatic index; IBW, initial body weight; SGR, specific growth rate; SLA, basal diet + 1.00% sodium lactate; SLA + AM, 1.00% sodium lactate diet + 2.5 mg/L total ammonia nitrogen, SR, survival rate; WGR, weight gain rate.

**Table 4:** Effects of sodium lactate on the body composition of yellow catfish under ammonia stress.

Items	Protein (% wet matter)	Lipid (% wet matter)	Ash (% wet matter)	Moisture (%)
Individual treatments				
CON	13.95	8.06	3.18	73.44
AM	14.16	8.17	2.93	72.59
SLA	13.23	7.66	3.08	73.62
SLA + AM	13.29	7.73	2.96	73.91
SEM	0.372	0.307	0.144	0.619
Main effect treatments				
No AM	13.59	7.86	3.13	73.53
AM	13.73	7.95	2.95	73.25
No SLA	14.06	8.12	3.06	73.02
SLA	13.26	7.70	3.02	73.77
*p*‐Value (two‐way ANOVA)				
AM	0.728	0.773	0.230	0.666
SLA	0.063	0.208	0.799	0.261
Interaction	0.842	0.955	0.642	0.383

*Note:* Values are presented as mean ± SEM, *n* = 3 replicates per treatment. Two‐way ANOVA was used to evaluate the main effects of AM, SLA, and their interaction. When the interaction effect was significant (*p* < 0.05), simple effect analysis followed by Duncan’s multiple range test was performed for pairwise comparisons. No significant interaction or main effects were detected for all parameters (*p* > 0.05), so post‐hoc pairwise comparisons and superscript letter marking were not performed. Protein, lipid and ash contents are expressed on a wet matter basis.

A significant interaction between ammonia stress and SLA was only observed for HSI (*p* = 0.03). No significant interactions were found for FBW, WGR, SGR, FCR, FI, CF, and SR (*p* > 0.05). Among all individual treatments, fish fed the SLA diet without ammonia stress exhibited the highest FBW (8.98 g), WGR (447.89%), and SGR (3.04%/day), while ammonia‐stressed fish fed the basal diet showed the poorest growth performance and the highest FCR (1.59). Fish in the SLA + AM group showed significantly improved growth and feed utilization compared with those in the AM group.

For whole‐body proximate composition, no significant main effects of ammonia stress or SLA, nor their interaction, were detected for crude protein, crude lipid, ash, and moisture contents (*p* > 0.05). All body composition parameters remained relatively stable across the four treatment groups.

### 3.2. Serum Biochemical Indices

Serum biochemical indices, glucose metabolism parameters, and SA content are presented in Tables 5 and 6.

**Table 5:** The effects of sodium lactate on serum biochemistry of yellow catfish under ammonia stress.

Items	TP (mg/mL)	TG (mmol/L)	TC (mmol/L)	ALT (U/L)	AST (U/L)	ALB (g/L)	BUN (mmol/L)
Individual treatments							
CON	17.57	2.34	2.08	5.96	8.07^b^	7.60	1.66^b^
AM	15.58	2.39	2.06	8.08	17.32^c^	4.92	2.37^c^
SLA	20.50	2.88	2.38	3.96	6.72^a^	9.17	1.31^a^
SLA + AM	18.83	2.15	2.20	4.68	8.40^ab^	6.93	1.50^ab^
SEM	0.461	0.272	0.130	0.414	0.408	0.387	0.085
Main effect treatments							
No AM	19.04	2.61	2.23	4.96	7.39	8.38	1.49
AM	17.20	2.27	2.13	6.38	12.86	5.92	1.94
No SLA	16.57	2.37	2.07	7.02	12.70	6.26	2.02
SLA	19.66	2.52	2.29	4.32	7.56	8.05	1.41
*p*‐Value (two‐way ANOVA)							
AM	0.004	0.251	0.486	0.009	<0.001	<0.001	0.001
SLA	<0.001	0.592	0.133	<0.001	<0.001	0.002	<0.001
Interaction	0.742	0.186	0.564	0.128	<0.001	0.580	0.015

*Note:* Values are presented as mean ± SEM, *n* = 3 replicates per treatment. Two‐way ANOVA was performed to assess the main effects of AM, SLA, and their interaction. When the interaction effect was significant (*p* < 0.05), simple effect analysis followed by Duncan’s multiple range test was performed for pairwise comparisons. Different lowercase superscript letters in the same row indicate significant differences among treatments (*p* < 0.05).

Abbreviations: ALB, albumin; ALT, alanine aminotransferase; AST, aspartate aminotransferase; BUN, blood urea nitrogen; TC, total cholesterol; TG, triglyceride; TP, total protein.

**Table 6:** Effects of sodium lactate on serum glucose metabolism parameters and ammonia content in juvenile yellow catfish under chronic ammonia stress.

Items	LDH (U/L)	LAC (mmol/L)	PYR (μmol/L)	GLU (mmol/L)	SA (μmol/L)
Individual treatments					
CON	367.83	1.83	0.15^b^	5.10	247.71
AM	364.34	1.56	0.11^a^	4.25	334.22
SLA	415.50	2.64	0.27^d^	7.26	178.10
SLA + AM	412.79	2.44	0.18^c^	5.81	259.82
SEM	10.846	0.048	0.008	0.134	15.022
Main effect treatments					
No AM	391.67	2.23	0.21	6.18	212.91
AM	388.56	2.00	0.14	5.03	297.02
No SLA	366.08	1.70	0.13	4.67	290.96
SLA	414.14	2.54	0.23	6.54	218.96
*p*‐Value (two‐way ANOVA)					
AM	0.782	0.001	<0.001	<0.001	0.001
SLA	0.002	<0.001	<0.001	<0.001	0.001
Interaction	0.972	0.478	0.014	0.055	0.877

*Note:* Values are presented as mean ± SEM, *n* = 3 replicates per treatment. Two‐way ANOVA was applied to examine the main effects of AM, SLA, and their interaction. When the interaction effect was significant (*p* < 0.05), simple effect analysis followed by Duncan’s multiple range test was performed for pairwise comparisons. Different lowercase superscript letters in the same row indicate significant differences among treatments (*p* < 0.05).

Abbreviations: GLU, glucose; LAC, lactate; LDH, lactate dehydrogenase; PYR, pyruvate; SA, serum ammonia.

Ammonia stress caused significant reductions in TP and ALB (*p* < 0.01), and significant elevations in ALT, AST, and BUN (*p* < 0.01). Dietary SLA supplementation significantly increased TP and ALB levels (*p* < 0.01), and decreased ALT, AST activities, and BUN content (*p* < 0.01). No significant effects of ammonia or SLA on TG and TC were observed (*p* > 0.05). Significant interactions between ammonia stress and SLA were detected for AST and BUN (*p* < 0.05). Post‐hoc analysis showed that AST activity was the highest in the AM group (17.32 U/L) and the lowest in the SLA group (6.72 U/L); SLA supplementation significantly mitigated the ammonia‐induced increase in AST (SLA + AM vs. AM, *p*  < 0.05). Similarly, the BUN content was significantly elevated by ammonia stress (AM: 2.37 mmol/L vs. CON: 1.66 mmol/L), and this elevation was effectively reversed by SLA supplementation (SLA + AM: 1.50 mmol/L).

For glucose metabolism parameters, ammonia stress significantly decreased LAC, PYR, and GLU contents (*p* ≤ 0.001), and significantly increased SA concentration (*p* = 0.001). No significant effect of ammonia on LDH activity was found (*p* > 0.05). Dietary SLA supplementation significantly increased LDH activity, LAC, PYR, and GLU contents (*p* < 0.01), and reduced SA level (*p* = 0.001). A significant interaction between ammonia stress and SLA was observed for PYR (*p* = 0.014). The PYR content was the highest in the SLA group (0.27 μmol/L) and the lowest in the AM group (0.11 μmol/L). SLA supplementation significantly increased PYR content in ammonia‐stressed fish (SLA + AM vs. AM, *p*  < 0.05).

### 3.3. Hepatic Antioxidant Enzyme Activities

Hepatic antioxidant indices are presented in Table 7. Ammonia stress significantly decreased the activities of SOD, CAT, and GSH content (*p* < 0.001), and increased MDA level (*p* < 0.001). T‐AOC showed no significant response to ammonia stress (*p* > 0.05). Dietary SLA supplementation significantly enhanced SOD, CAT activities, and GSH content (*p* < 0.001), and reduced MDA accumulation (*p* = 0.001). No significant main effect of SLA on T‐AOC was detected (*p* > 0.05).

**Table 7:** Effects of sodium lactate on liver antioxidant enzymes of yellow catfish under ammonia stress.

Items	T‐AOC (U/mgprot)	SOD (U/mgprot)	CAT (U/mgprot)	GSH (μmol/gprot)	MDA (nmol/gprot)
Individual treatments					
CON	2.37	93.30^b^	75.38	128.76	47.53
AM	2.00	53.69^a^	56.07	104.48	66.57
SLA	2.26	105.08^c^	86.39	161.96	35.69
SLA + AM	2.18	84.53^b^	76.27	130.96	52.13
SEM	0.184	2.820	2.586	1.855	2.712
Main effect treatments					
No AM	2.32	99.19	80.89	145.36	41.61
AM	2.09	69.11	66.17	117.72	59.35
No SLA	2.19	73.50	65.73	116.62	57.05
SLA	2.22	94.81	81.33	146.46	43.91
*p*‐Value (two‐way ANOVA)					
AM	0.257	<0.001	<0.001	<0.001	<0.001
SLA	0.863	<0.001	<0.001	<0.001	0.001
Interaction	0.468	0.010	0.114	0.108	0.644

*Note:* Values are presented as mean ± SEM, *n* = 3 replicates per treatment. Two‐way ANOVA was used to test the main effects of AM, SLA, and their interaction. When the interaction effect was significant (*p* < 0.05), simple effect analysis followed by Duncan’s multiple range test was performed for pairwise comparisons. Different lowercase superscript letters in the same row indicate significant differences among treatments (*p* < 0.05).

Abbreviations: CAT, catalase; GSH, glutathione; MDA, malondialdehyde; SOD, superoxide dismutase; T‐AOC, total antioxidant capacity.

A significant interaction between ammonia stress and SLA was found for SOD activity (*p* = 0.01). Multiple comparisons revealed that SOD activity was the highest in the SLA group (105.08 U/mgprot) and the lowest in the AM group (53.69 U/mgprot). SLA supplementation effectively alleviated the ammonia‐induced suppression of SOD activity, with the SLA + AM group showing significantly higher SOD activity than the AM group (*p* < 0.05).

### 3.4. Intestinal Digestive Enzyme Activities

Intestinal digestive enzyme activities are shown in Table 8. Ammonia stress significantly decreased lipase and pepsin activities (*p* < 0.01), while amylase activity was not significantly affected (*p* > 0.05). Dietary SLA supplementation significantly increased lipase and pepsin activities (*p* ≤ 0.001), with no significant effect on amylase (*p* > 0.05).

**Table 8:** The effects of sodium lactate on intestinal digestive enzymes of yellow catfish under ammonia stress.

Items	Lipase (U/gprot)	Amylase (U/mgprot)	Pepsin (U/mgprot)
Individual treatments			
CON	31.92	0.82	9.99^b^
AM	25.10	0.74	6.32^a^
SLA	40.82	0.80	14.98^c^
SLA + AM	32.84	0.82	9.23^b^
SEM	1.629	0.058	0.392
Main effect treatments			
No AM	36.37	0.81	12.49
AM	28.97	0.78	7.78
No SLA	28.51	0.78	8.16
SLA	36.83	0.81	12.11
*p*‐Value (two‐way ANOVA)			
AM	0.002	0.674	<0.001
SLA	0.001	0.631	<0.001
Interaction	0.734	0.388	0.029

*Note:* Values are presented as mean ± SEM, *n* = 3 replicates per treatment. Two‐way ANOVA was applied to evaluate the main effects of AM, SLA, and their interaction. When the interaction effect was significant (*p* < 0.05), simple effect analysis followed by Duncan’s multiple range test was performed for pairwise comparisons. Different lowercase superscript letters in the same row indicate significant differences among treatments (*p* < 0.05).

A significant interaction between ammonia stress and SLA was observed for pepsin activity (*p* = 0.029). Pepsin activity was the highest in the SLA group (14.98 U/mgprot) and the lowest in the AM group (6.32 U/mgprot). Under ammonia stress, SLA supplementation restored pepsin activity to a level comparable to the control group (SLA + AM vs. CON, *p*  > 0.05).

### 3.5. TCA Cycle Gene Expression

**Figure 1:**
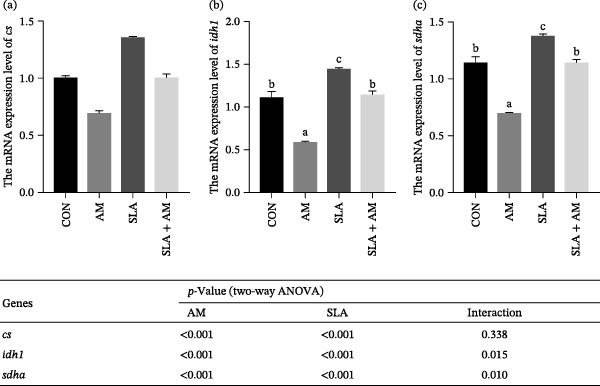
Effects of dietary sodium lactate (SLA) on mRNA expression of key TCA cycle genes in the intestine of juvenile yellow catfish under ammonia stress (AM). (a) *cs*, (b) *idh1*, (c) *sdha*. Data are mean ± SEM (*n* = 3). Two‐way ANOVA was used to test the main effects of AM, SLA, and their interaction. When the interaction effect was significant (*p* < 0.05), simple effect analysis followed by Duncan’s multiple range test was performed for pairwise comparisons. Different lowercase letters on bars denote significant differences among treatments (*p* < 0.05).

The expression profiles of key TCA cycle genes in the intestine (*cs*, *idh1*, and *sdha*) are illustrated in Figure 1. Ammonia stress significantly downregulated the mRNA expression of *cs*, *idh1*, and *sdha* (*p* < 0.001). Dietary SLA supplementation significantly upregulated the expression of all three genes (*p* < 0.001).

Significant interactions between ammonia stress and SLA were detected for *idh1* and *sdha* (*p* < 0.05), but not for *cs* (*p* > 0.05). Post‐hoc analysis indicated that the expression levels of *idh1* and *sdha* were the lowest in the AM group and the highest in the SLA group. SLA supplementation significantly reversed the ammonia‐induced downregulation of *idh1* and *sdha* (SLA + AM vs. AM, *p*  < 0.05).

### 3.6. Glucose Metabolism Related Genes

**Figure 2:**
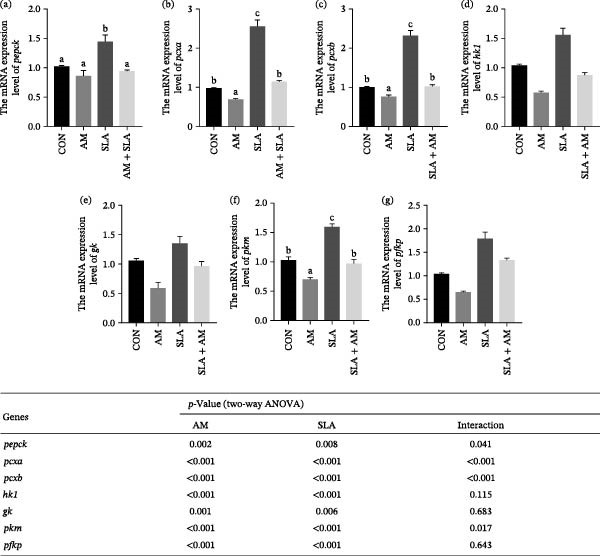
Effects of dietary sodium lactate (SLA) on mRNA expression of key hepatic glucose metabolism genes in juvenile yellow catfish under ammonia stress (AM). (a) *pepck*, (b) *pcxa*, (c) *pcxb*, (d) *hk1*, (e) *gk*, (f) *pkm*, (g) *pfkp*. Data are mean ± SEM (*n* = 3). Two‐way ANOVA was used to test the main effects of AM, SLA, and their interaction. When the interaction effect was significant (*p* < 0.05), simple effect analysis followed by Duncan’s multiple range test was performed for pairwise comparisons. Different lowercase letters on bars denote significant differences among treatments (*p* < 0.05).

The expression of glucose metabolism‐related genes in the liver is shown in Figure 2. Ammonia stress significantly downregulated the mRNA expression of all detected genes, including *pepck*, *pcxa*, *pcxb*, *hk1*, *gk*, *pkm*, and *pfkp* (*p* < 0.01). Dietary SLA supplementation significantly upregulated the expression of these genes (*p* < 0.01).

Significant interactions between ammonia stress and SLA were observed for *pepck*, *pcxa*, *pcxb*, and *pkm* (*p* < 0.05), while no interactions were found for *hk1*, *gk*, and *pfkp* (*p* > 0.05). For genes with significant interactions, the lowest expression levels were observed in the AM group, and SLA supplementation effectively restored their expression under ammonia stress. The *pcxa* and *pcxb* genes showed the most pronounced interactive effect (*p* < 0.001).

### 3.7. Growth Related Genes

**Figure 3:**
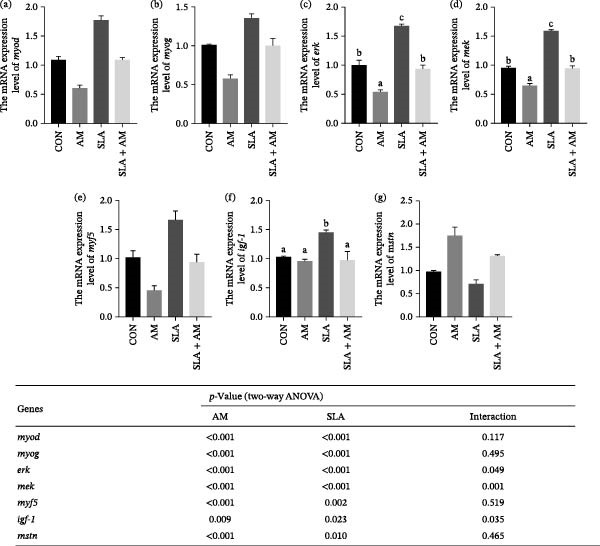
Effects of dietary sodium lactate (SLA) on mRNA expression of muscle growth‐related genes in juvenile yellow catfish under ammonia stress (AM). (a) *myod*, (b) *myog*, (c) *erk*, (d) *mek*, (e) *myf5*, (f) *igf-1*, (g) *mstn*. Data are mean ± SEM (*n* = 3). Two‐way ANOVA was used to test the main effects of AM, SLA, and their interaction. When the interaction effect was significant (*p* < 0.05), simple effect analysis followed by Duncan’s multiple range test was performed for pairwise comparisons. Different lowercase letters on bars denote significant differences among treatments (*p* < 0.05).

The expression of muscle growth‐related genes is presented in Figure 3. Ammonia stress significantly downregulated the mRNA expression of positive growth regulators (*myod*, *myog*, *erk*, *mek*, *myf5*, and *igf-1*) (*p* < 0.01), and significantly upregulated the negative growth regulator myostatin (*mstn*) (*p* < 0.001). Dietary SLA supplementation significantly upregulated *myod*, *myog*, *erk*, *mek*, *myf5*, and *igf-1* (*p* < 0.05), and downregulated *mstn* expression (*p* < 0.05).

Significant interactions between ammonia stress and SLA were detected for *erk*, *mek*, and *igf-1* (*p* < 0.05), whereas no interactions were found for *myod*, *myog*, *myf5*, and *mstn* (*p* > 0.05). Post‐hoc test showed that the expression of *erk*, *mek*, and *igf-1* was significantly lower in the AM group than in the CON group, and SLA supplementation significantly elevated their expression in ammonia‐stressed fish.

## 4. Discussion

### 4.1. Growth, Digestive Function and Serum Biochemistry

Chronic ammonia exposure inhibits fish growth and impairs digestive function, which is a core constraint limiting yellow catfish production in high‐density farming [16]. In the present study, chronic ammonia stress significantly decreased FBW, WGR, and FCR of juvenile yellow catfish, in agreement with earlier studies on common carp (*Cyprinus carpio*) [17] and Japanese seabass (*Lateolabrax japonicus*) [18]. Dietary 1.00% SLA supplementation effectively alleviated ammonia‐induced growth retardation, which aligns with the growth‐promoting effects of lactate salts reported in beluga sturgeon (*Huso huso*) and common carp [19, 20].

The improved feed efficiency is likely associated with enhanced digestive enzyme activities. Ammonia stress reduced intestinal lipase and pepsin activities, and SLA supplementation restored these enzyme activities. As an organic acid salt, SLA may optimize digestive function by lowering intestinal pH and improving nutrient assimilation [21], analogous to the effects of sodium butyrate in black sea bream [22]. Since FI showed no significant difference across groups, the growth benefit of SLA is driven by improved nutrient utilization rather than increased feed consumption.

Serum biochemical parameters can serve as direct indicators of overall health condition, liver function, and metabolic status in fish [14]. Ammonia exposure induced typical hepatocellular injury signatures (elevated ALT and AST and reduced TP and ALB) and impaired ammonia excretion (elevated SA and BUN), consistent with the classic ammonia toxicity pattern in fish [23]. SLA supplementation attenuated these alterations, suggesting that SLA facilitates hepatic ammonia detoxification and reduces hepatotoxicity, which may contribute to improved ammonia tolerance.

### 4.2. Hepatic Antioxidant Capacity

Oxidative stress is a well‐documented secondary effect of ammonia toxicity, driven by excessive reactive oxygen species (ROS) accumulation [24]. In this study, ammonia stress suppressed hepatic SOD and CAT activities and GSH content while increasing the MDA level, indicating lipid peroxidation damage. This pattern is consistent with previous findings in guppies (*Poecilia reticulata*), where ammonia stress disrupted oxidation‐antioxidant homeostasis and impaired antioxidant compensatory capacity [25]. SLA supplementation reversed these changes, suggesting that SLA strengthens hepatic antioxidant defense to counteract ammonia‐induced oxidative injury.

The underlying antioxidant mechanism of SLA in fish remains to be clarified. Mammalian studies indicate that lactate may act as a signaling molecule to activate antioxidant pathways, and NADH generated during lactate metabolism may supply reducing power for antioxidant systems [26, 27]. Metabolic regulation of mitochondrial ROS production by SLA, as reported in Nile tilapia [13], may also contribute to this protective effect.

### 4.3. Glucose Metabolism and Energy Homeostasis

Another major toxic effect of ammonia stress in fish is the disruption of systemic energy metabolic homeostasis [28]. Our data showed that ammonia stress reduced serum GLU, LAC, and PYR concentrations and downregulated hepatic mRNA expression of key genes in glycolysis, gluconeogenesis, and the TCA cycle. These changes suggest that chronic ammonia stress broadly inhibits glucose catabolism and may compromise the mitochondrial energy supply.

SLA supplementation increased serum LAC and PYR levels and upregulated the expression of genes in the glucose metabolic pathways. Specifically, SLA may enter hepatocytes and be converted to pyruvate, which may on one hand activate the glycolytic pathway via upregulating key genes such as *gk*, *hk1*, *pkm*, and *pfkp* [29, 30], and on the other hand provide carbon skeletons for the TCA cycle [31]. This regulatory pattern suggests that SLA may replenish metabolic substrates and alleviate the energy deficit induced by ammonia stress, consistent with the model proposed in Nile tilapia [13].

Notably, these inferences are based solely on serum metabolites and mRNA expression. Whether such transcriptional changes translate to enhanced ATP production, mitochondrial respiration, or metabolic flux remains unconfirmed, requiring further functional validation.

### 4.4. Muscle Growth Regulation

Under ammonia stress, fish reallocate energy from somatic growth to detoxification and damage repair, which is a major cause of growth retardation. The improved energy status associated with SLA supplementation may allow greater allocation of nutrients to muscle growth.

At the molecular level, SLA reversed the ammonia‐induced downregulation of myogenic regulatory genes and upregulation of the growth suppressor *mstn*. Previous research in Nile tilapia suggested that lactate may activate the mammalian target of rapamycin (mTOR) signaling pathway, which interacts with *igf-1* and MAPK cascades to regulate muscle growth [13]. Our gene expression data are consistent with this model, and similar regulatory effects of the nutrient‐activated mTOR pathway on muscle growth have also been reported in hybrid catfish (*Pelteobagrus vachelli* × *Leiocassis longirostris*) [32]. However, protein‐level validation of the signaling pathway is still lacking.

### 4.5. Study Limitations

Only one inclusion level of SLA was evaluated, and a graded dose design would have provided greater insight into the dose–response relationship and optimal dosage for juvenile yellow catfish. Additionally, incorporating other assessments, such as gut microbiome profiling, histopathological examination, and protein‐level validation of signaling pathways, would have further substantiated the mechanistic inferences.

Despite these constraints, this is the first study to demonstrate the protective effect of dietary SLA against chronic ammonia stress in yellow catfish and reveal its underlying pathways. Our findings support SLA as a functional feed additive for ammonia stress mitigation in intensive aquaculture and provide a basis for further mechanistic research.

## 5. Conclusion

In conclusion, dietary SLA improved the growth performance and antioxidant status of juvenile yellow catfish and alleviated multiple adverse effects induced by chronic ammonia stress. This study suggests that SLA can serve as a promising functional feed additive to counteract ammonia toxicity in intensive yellow catfish farming.

## Author Contributions

**Ruiqiong Zhang**: writing ‐ original draft, methodology, investigation. **Ming Li**: writing ‐ review and editing, supervision, project administration, funding acquisition, conceptualization. **Yanjie Tang, Ying Yan, and Xuan Chen**: methodology, investigation, project administration. **Haibo Jiang and Muzi Zhang**: validation, resources, project administration.

## Funding

This work was supported by the National Natural Science Foundation of China (Grants 32473130 and 32573483) and the Natural Science Foundation of Guizhou Province of China (Grant MS[2026]201).

## Conflicts of Interest

The authors declare no conflicts of interest.

## Data Availability

The data will be made available upon request.
